# Molecular basis of Mitomycin C enhanced corneal sensory nerve repair after debridement wounding

**DOI:** 10.1038/s41598-018-35090-3

**Published:** 2018-11-16

**Authors:** Mary Ann Stepp, Sonali Pal-Ghosh, Gauri Tadvalkar, Luowei Li, Stephen R. Brooks, Maria I. Morasso

**Affiliations:** 1 Department of Anatomy and Cell Biology, George Washington University School of Medicine and Health Sciences, Washington, DC 20037 USA; 2 Department of Ophthalmology, George Washington University School of Medicine and Health Sciences, Washington, DC 20037 USA; 3 Laboratory of Cancer Biology and Genetics, NCI, NIH, Bethesda, MD 20892 USA; 4 Biodata Mining and Discovery Section, National Institute of Arthritis and Musculoskeletal and Skin Diseases, National Institutes of Health, Bethesda, MD 20892 USA; 5 Laboratory of Skin Biology, National Institute of Arthritis and Musculoskeletal and Skin Diseases, National Institutes of Health, Bethesda, MD 20892 USA

## Abstract

The ocular surface is covered by stratified squamous corneal epithelial cells that are in cell:cell contact with the axonal membranes of a dense collection of sensory nerve fibers that act as sentinels to detect chemical and mechanical injuries which could lead to blindness. The sheerness of the cornea makes it susceptible to superficial abrasions and recurrent erosions which demand continuous regrowth of the axons throughout life. We showed previously that topical application of the antibiotic and anticancer drug Mitomycin C (MMC) enhances reinnervation of the corneal nerves and reduces recurrent erosions in mice via an unknown mechanism. Here we show using RNA-seq and confocal imaging that wounding the corneal epithelium by debridement upregulates proteases and protease inhibitors within the epithelium and leads to stromal nerve disruption. MMC attenuates these effects after debridement injury by increasing serpine1 gene and protein expression preserving L1CAM on axon surfaces of reinnervating sensory nerves. These data demonstrate at the molecular level that gene expression changes in the corneal epithelium and stroma modulate sensory axon integrity. By preserving the ability of axons to adhere to corneal epithelial cells, MMC enhances sensory nerve recovery after mechanical debridement injury.

## Introduction

Light passes through the cornea on its way to the retina. The cornea functions as the primary refractive surface of the eye. Light is refracted while passing from air to the liquid interface formed by the tear film. This directs light waves to the macula and permits maximal visual acuity. The uniform spreading of the tear film on the cornea is vital for vision and in turn, healthy corneal epithelial cells are vital for maintaining a stable tear film. Injuries that induce corneal epithelial erosions and stromal scarring destabilize the tear film and impair vision^1^. To minimize the risk of infections and restore the eyes refractive power, injuries to the cornea evolved mechanisms to allow them to heal quickly.

Corneal epithelial cells are unique in their ability to heal injuries rapidly^1^ and to preserve and support a dense collection of sensory nerves, referred to as intraepithelial corneal nerves (ICNs) that are present on the ocular surface^2^. Recent studies indicate that ICN function is reduced in dry eye disease and studies are currently underway to determine whether loss of ICN function is a cause or consequence of dry eye disease^3–5^.

Previously we showed that treating mouse corneas injured by 1.5 mm debridement topically with the antibiotic and anti-cancer drug Mitomycin C (MMC), soon after reepithelialization is completed, enhances sensory axon recovery and reduces erosion frequency significantly^6^. MMC is used clinically in ophthalmology to reduce scarring after surgical procedures including refractive surgery^7–10^. Adult epidermal and corneal epithelial progenitor cells cease proliferating and terminally differentiate *in vitro* unless seeded onto “feeder layers” of MMC-treated fibroblasts that sustain their proliferation and reduce their differentiation. The feeder layers allow the epithelial progenitor cells to be used for regeneration of the skin and cornea after severe injury or pathology^11^. How MMC functions to both reduce scarring and to maintain epithelial progenitor cells in their dedifferentiated state is not clear. Here, after demonstrating that MMC treatment enhances reinnervation of the ICNs when applied at the time of injury, we use RNA-seq transcriptomic analyses to determine the mechanisms underlying the ability of MMC to impact corneal wound resolution and ICN reinnervation. Insights gained from these RNA-seq data allow us to demonstrate the involvement of secreted proteases in corneal axon homeostasis and reinnervation after injury and the importance of stromal nerve integrity in these processes. These data also allow us to propose that MMC treatment of corneas with injuries that sever nerves but do not induce reepithelialization will delay reinnervation and we test the hypothesis experimentally. These data provide a rich resource to the community to understand how reepithelialization and reinnervation occur rapidly and efficiently in the cornea.

## Methodology

### Animals

All studies performed using mice were approved by the George Washington University Medical Center Institutional Animal Care and Use Committee. These studies comply with all relevant guidelines. In addition, they comply with the Association for Research in Vision and Ophthalmology (ARVO) Statement for the Use of Animals in Vision and Ophthalmic Research (https://www.arvo.org/About/policies/statement-for-the-use-of-animals-in-ophthalmic-and-vision-research). For all wounding experiments, 7w–8w male BALB/c mice were ordered from Charles River (Frederick MD). Mice were anesthetized with ketamine/xylazine and a topical anesthetic applied to their ocular surface as described previously^12^. Wounding was bilateral. For trephine wounds, a 1.5 mm dulled trephine was used to demarcate the wound area. For debridement wounds, the epithelial cells within the 1.5 mm area defined by the trephine were removed using a dulled blade. After wounding, erythromycin ophthalmic ointment was applied, and mice were allowed to recover. Mice were sacrificed 18 hr after wounding for debridement or at 2d, 3d, 4d, 7d, 14d, 28d and 42d for trephine studies. The number of corneas assessed for each study are indicated within each figure legend. Since earlier studies show that differences between axon density in left and right corneas are similar to differences between individual mice of the same age and gender, corneas are considered as independent variables. The number of mice used for each study is 1/2 the number of corneas used.

### Mitomycin C (MMC) Treatment

For RNA-seq and immunofluorescence studies, corneas received one topical application (20 μl per eye) of 0.02% Mitomycin-C (#3258, Tocris) dissolved in PBS at the time of wounding (MW) or 18 hr prior to sacrifice in the control unwounded mice (MC). After 2 minutes, excess solution was removed with a Kimwipe and by blinking. For trephine wounded corneas, the same volume (20 μl/eye) and concentration of MMC was applied at the time of and 18 hr after wounding.

### Aprotinin Treatment

The concentration of aprotinin (10,000 KIU/ml) was the same as that used in rabbit cornea wound healing studies^13^. Aprotinin (20 ul) is applied for 2 min either alone, before, or after 2 min of MMC treatment as described above. Between the 2 applications, a Kimwipe is used to remove excess solution from the surface of the cornea. Mice are sacrificed 18 hr after treatment.

### IF studies

For immunofluorescence (IF) studies, tissues were pooled for each time point and wound type and fixed immediately after euthanization in a paraformaldehyde-containing fixative (1x PBS, 1% formaldehyde, 2 mM MgCl2, 5 mM EGTA, 0.02% NP-40) for 1 hr and 15 min at 4 °C, followed by 2 washes for 10 min each in 1x PBS containing 0.02% NP40 at room temperature. Tissues were then placed in 4:1 methanol:DMSO solution for 2 hr at −20 °C and then stored in 100% methanol at −20 °C until used for whole mount staining studies.

Whole mount staining has been described previously^6^. In brief, the back of eyes fixed as described above are removed, along with the lens and iris, and 4 incisions are made in each quadrant of the cornea. Following graded triton-methanol treatment and 2 hr in blocking buffer, corneas are incubated overnight with primary antibody diluted in blocking buffer at 4 °C. The next day, the tissues are washed five times with PBS (1 hr each) and 0.02% Tween 20 (PBST), blocked for 2 hr, and then incubated with secondary antibody diluted in blocking buffer overnight at 4 °C. The following day, eyes are washed three times with PBST for 1 hr each, followed by nuclear staining with DAPI (Thermo fisher; #46190) for 5 minutes, and washed with distilled water. The corneas are oriented epithelial side-up using mounting media (Fluoromount G; Electron Microscopy Sciences; #17984-25) and coverslipped.

### Antibodies

Corneas were stained with the following antibodies: βIII tubulin (Biolegend, #801201), α3^14^, L1CAM (Millipore, #MAB5272), Notch 1 (Abcam, #ab52627), P21 (Santa Cruz, #SC6246), PLK1 (Santa Cruz, #SC17783), Iba1 (Wako, #019-19741), Serpine2 (Proteintech, #11303-1-AP). Appropriate secondary DyLite 488, 594, and 647 antibodies from Jackson ImmunoResearch were used for immunolabeling.

### Microscopy

Confocal microscopy was performed at the GW Nanofabrication and Imaging Center at the George Washington University Medical Center. For Sholl analysis, images were acquired using the Zeiss Cell Observer Z1 spinning disk confocal microscope (Carl Zeiss, Inc., Thornwood, NY, USA), equipped with ASI MS-2000 (Applied Scientific Instrumentation, Eugene, OR, USA) scanning stage with z-galvo motor, and Yokogawa CSU-X1 spinning disk. A multi-immersion 25x/0.8 objective lens, LCI Plan-Neofluor, was used for imaging, with oil immersion. Evolve Delta (Photometrics, Tucson, AZ, USA) 512 × 512 EM-CCD camera was used as detector (80-msec exposure time). A diode laser emitting at 568 nm was used for excitation (54% power). Zen Blue software (Carl Zeiss, Inc.) was used to acquire the images, fuse the adjacent tiles, and produce maximum intensity projections. The adjacent image tiles were captured with overlap to ensure proper tiling. All images were acquired using the same intensity settings. Sholl analysis was performed using ImageJ (https://imagej.net/Sholl_Analysis) as described previously^12^. In brief, in Sholl analysis, a ring consisting of several concentric circles is placed onto an image and the number of times an axon intersects with one or more of the rings of the circle is quantified. For these studies, three concentric circles are placed at the center of the cornea and one each at the four corners at the periphery of each 21-panel image acquired.

For high-resolution immunofluorescence imaging, a confocal laser-scanning microscope (Zeiss 710) was used to image the localization of Alexa Fluor 488 (argon laser; 488 nm laser line excitation; 495/562 emission filter), Alexa Fluor 594 (561 diode laser; 594 nm laser line excitation; 601/649 emission filter) and Alexa Fluor 647 (633 Diode laser; 647 nm laser line excitation; 671/759 emission filter). Optical sections (*z* = 0.5 μm or 1 μm) were acquired sequentially with a 63x objective lens. 3D images were rotated to generate cross section views using Volocity software (Version 6.3, Perkin Elmer). High-resolution images are presented either as cross sections projected through the length of the acquired image or en face projections. Images are obtained using the same confocal laser settings and the same intensity settings in Volocity to permit valid comparisons for each antibody assessed.

### Quantification

The area of the remaining wound was measured using ImageJ. The free hand tool was used to trace around the open wound and area measured by selecting ‘Area’ in the ‘Set Measurement’ tool. For W and MW corneas, 20 corneas are assessed from 10 mice. The intensity of staining for Notch1, p21, and Plk1 are quantified in a minimum of 4 corneas using 10x confocal images. Color images are converted to 8 bit B/W images, an 80 mm × 80 mm box is drawn on the image using the rectangular selection tool and pixel intensity is measured using Analyze - Set Measurement - Mean Gray value on the tool bar. APC size is measured using 63x confocal images; the straight line tool is used to join the protruding/projecting tips of the APC to demarcate the area and numbers obtained by selecting Analyze - Set Measurement-Area on the tool bar as described^15^. APC number is determined by counting the numbers of APC in 10x images acquired from all 4 quadrants of 4 eyes for each variable assessed. The lengths of the L1CAM+ and βIII tubulin+ ICNs and stromal nerves are assessed using NeuronJ. Images are converted to 8 bit. Using the ‘Add tracing’ tool from the tool bar, the lengths of the nerves are traced in 4 corneas per variable.

### RNA-seq

For RNA-seq studies, epithelium was scraped using a dulled blade and frozen immediately in liquid nitrogen. Epithelium from 4 corneas were pooled into a single sample. After limbal to limbal debridement, eyes were removed, corneas dissected from the retina, lens, and iris and the limbal rims removed to yield clear stromal buttons. Stromal buttons from 4 corneas were pooled into a single sample. For both epithelium and stroma, 4 samples (from 16 corneas) were assessed per variable tested (C, MC, W, MW). RNA was extracted from corneal epithelium and stromal buttons using Arcturus Picopure RNA isolation kit (Applied Biosystems; #12204-01) according to the manufacturer’s instructions. mRNA expression profiling was performed in the NIAMS Genome Core Facility at the NIH. Single end, 50 base reads were mapped to the mouse genome mm10 using TopHat 2.1.0 (http://tophat.cbcb.umd.edu). Expression values [reads per kilobase of transcript per million mapped reads (RPKM)] and fold changes were calculated and analyzed with the Partek Genomics Suite (http://www.partek.com). Analysis of variance (ANOVA) comparisons, False Discovery Rate (q-value) and Principal Components were calculated and analyzed with the Partek Genomics Suite 6.6 (http://www.partek.com). ANOVA was performed to compare the corneal wound healing process with/without MMC treatment (W/C, MC/C, MC/MW, MW/W). Only genes with robust expression (RPKM ≥ 1in at least one sample) were considered further. ANOVA results were filtered considering a significance value of q < 0.05 and an absolute fold change ≥2 or as noted in the corresponding figures. Gene Ontology (GO) terms were obtained via Gene Ontology Consortium (http://www.geneontology.org) using Enrichment Analysis and selecting biological processes and Mus musculus. Diseases and Functions terms and Upstream Regulators analysis were generated with IPA (Ingenuity Systems, www.ingenuity.com). Unless otherwise indicated, heat maps depict relative expression of each gene maximum and minimum log2 (RPKM) and were created using Morpheus (Broad Institute).

From each unwounded corneal epithelium harvested by limbal to limbal debridement we isolate 0.7-0.9 ng of RNA; wounded corneas contain 0.5–0.7 ng/RNA per cornea. RNA isolated from epithelial tissues derives primarily from corneal epithelial cells with minor contributions from resident immune cells and sensory axons. From each stromal button, we obtain ~10-fold less RNA or 0.07–0.09 ng per cornea. Stromal RNA includes RNA from corneal endothelial cells, resident stromal cells (keratocytes, immune cells, Schwann cells), as well as RNA from corneal epithelial cells lysed during debridement that remains associated with the stroma. GO analyses of RNA-seq data from stromal RNA generate ontology terms found in paired epithelial gene lists (data not shown). To address this issue, genes up or down regulated 2-fold or greater in epithelial gene lists are removed from paired stromal gene lists to generate enriched stromal gene lists. The number of 2-fold up and downregulated genes for the epithelium and stroma for all 4 comparisons (W:C, MC:C, MW:MC, and MW:W) are in Supplementary Table 1. Table 2 is an Excel file including the 2-fold up and downregulated genes in the epithelium, stroma, and enriched stroma data sets for all four comparisons including Probeset IDs, q values, and fold changes.

### Statistical analyses

Quantitative data are presented as mean ± standard error of the mean. All data were analyzed using the t-test for comparisons made between 2 variables or with one-way ANOVA for multiple variables using GraphPad Prism, Version 6 (GraphPad Software, Inc. San Diego, CA). When variances are significantly different, non-parametric testing is performed. For non-parametric testing. when only 2 groups are compared, the Mann-Whitney test is used; when more than 2 groups are compared, the Kruskal-Wallis test is used. A p value less than or equal to 0.05 was considered statistically significant. The type of statistical test used is indicated in each figure legend.

### Accession numbers

Completed RNA-Seq data has been deposited in the Gene Expression Omnibus (GEO) site. The accession number for these data is GSE115355.

## Results

### MMC treatment delays reepithelialization but enhances INT reinnervation and attenuates wound-mediated L1CAM shedding

**Figure 1:**
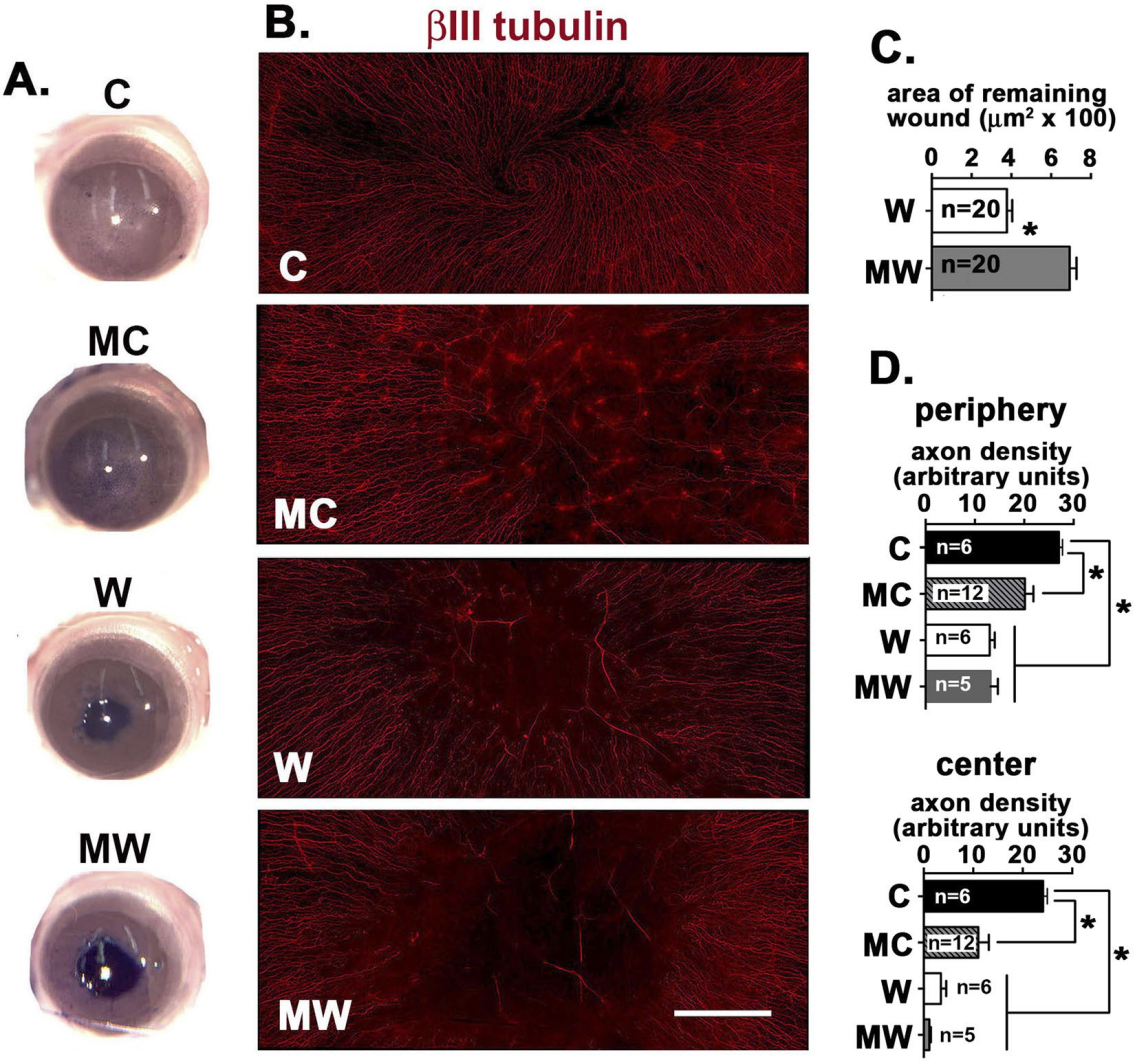
MMC treatment delays reepithelialization but enhances axon extension. (**A**) Control (C), corneas treated with MMC 18 hr prior to sacrifice (MC), 18 hr wounded without (W) and after MMC treatment at the time of injury (MW) were stained with a vital dye (Richardson Stain). (**B**) Representative en face confocal images from C, MC, W, and MW corneas stained to localize the intraepithelial corneal nerves using an antibody against βIII tubulin. (**C**) Quantitative assessment of the area of the remaining wound for 20 corneas for W and MW stained as described above in (**A**). The area of MW is significantly greater than W determined using the unpaired t-test with a p value less than or equal to 0.05. (**D**) Corneas stained with antibodies to βIII tubulin (**B**) were subjected to Sholl analysis to determine axon density at the center and periphery. Significance was determined using the non-parametric Kruskal-Wallis test for groups with different standard deviations; groups with p values less than or equal to 0.05 are considered significant. Magnification bar in B = 100 μm.

In response to 1.5 mm debridement injury, corneal epithelial cells migrate as a sheet; leading edge cells do not proliferate but cells at the corneal periphery increase their rate of proliferation^1^. Previously, we showed using a mouse model for recurrent epithelial corneal erosions that two topical MMC treatments applied after reepithelialization is complete enhances wound resolution and reinnervation^6^. To determine whether a single MMC treatment at the time of debridement injury impacts reepithelialization and reinnervation, we first assess the area of the remaining wound 18 hr after wounding in standard of care treated wounded corneas (W) and in wounded corneas treated with MMC at the time of wounding (MW). The vital dye Richardson stain was applied to the corneas after sacrifice and images acquired to allow quantification of the area of the wound using ImageJ. Unwounded corneas treated with MMC 18 hr before sacrifice (MC) were also assessed along with controls (C). Ocular surfaces are stained with a vital dye excluded from corneas with intact epithelial barriers. Representative images of corneas are shown in Fig. 1A; data for 20 W and MC corneas were quantified and show a significant delay in wound closure for MMC treated corneas in Fig. 1C.

After assessing wound areas, corneas were fixed and used for whole mount imaging to assess the localization of βIII tubulin, a neuronal specific tubulin that localizes to axons (Fig. 1B); axon density at the corneal periphery and center were quantified as described previously^12^. Data show that axon density is less in MC corneas compared to C at both the center and periphery (Fig. 1D). By 18 hr after wounding, reepithelialization is incomplete and at the center, ICNs are not detected. At the periphery, no differences in axon density are seen between W and MW corneas (Fig. 1D).

**Figure 2:**
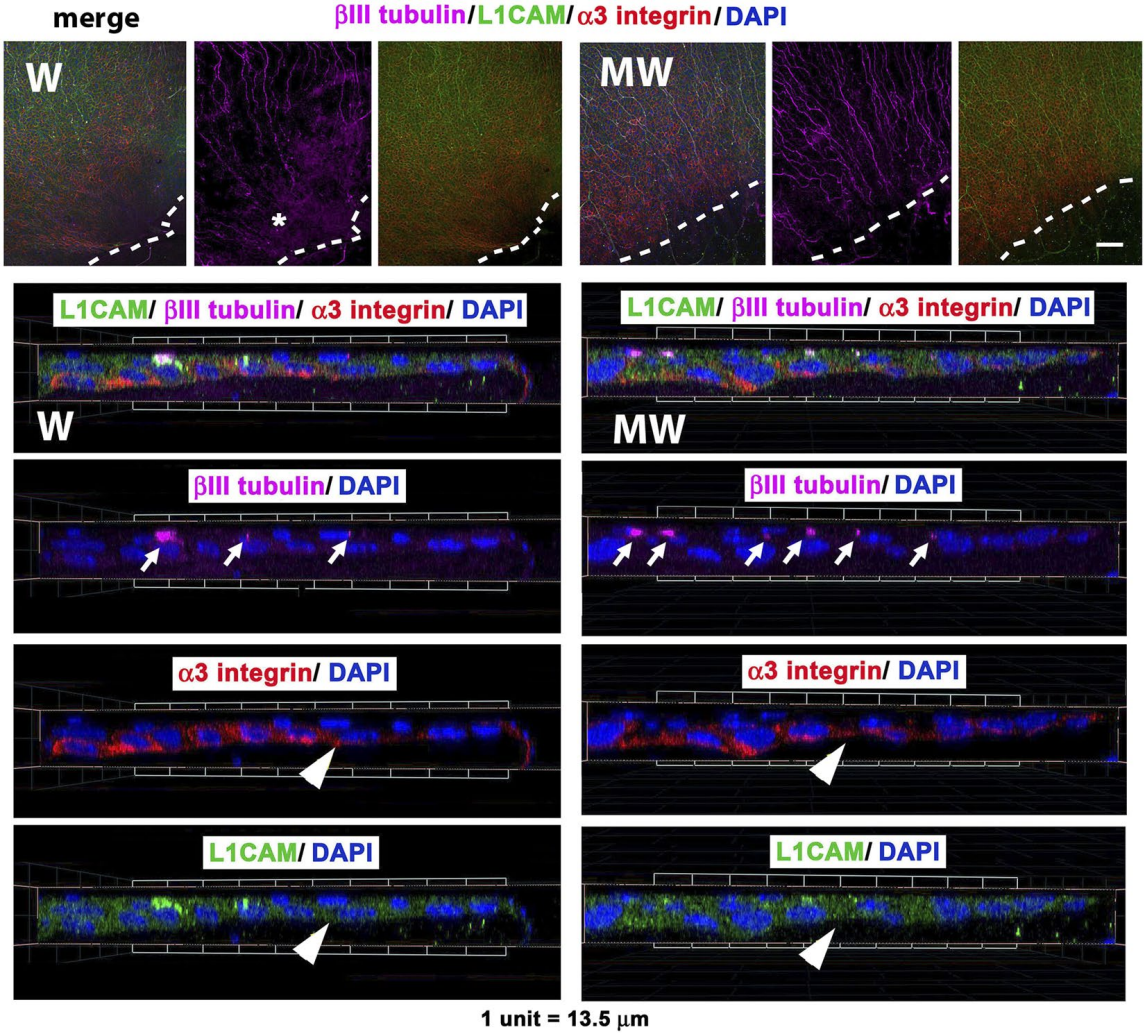
βIII tubulin^+^L1CAM^+^ axons extend closer to the leading edge in wounded MMC treated corneas. Representative en face confocal images obtained from the leading edges of W and MW corneas stained with antibodies to L1CAM (green), βIII tubulin (magenta), α3 integrin (red), and DAPI (blue) are presented. The dashed line indicates the leading edge. The asterisk highlights a site in the W cornea where axons are degraded and βIII tubulin staining is diffuse. Confocal images were also used to obtain 3D images that were rotated to generate cross sectional views of W and MW leading edges with migration proceeding from left to right; shown is the merger of all four colors followed by βIII tubulin/DAPI, α3 integrin/DAPI, and L1CAM/DAPI. Arrows show axons near the leading edge in W and MW corneas; arrowheads show α3 integrin and epithelial L1CAM are expressed at higher levels behind the leading edge. Magnification bar in the enface image = 50 μm.

Corneas were processed to reveal the localization of βIII tubulin, L1CAM, and α3 integrin in wounded (W) and MMC-treated wounded (MW) corneas 18 hr after injury. L1CAM is an integral membrane glycoprotein that localizes to sites of axon:axon and cell:axon adhesion^16,17^. α3 integrin is expressed by skin and corneal epithelial basal cell layers and mediates adhesion between cells as well as to laminin in the basement membrane^18,19^. Data show that in both W and MW corneas, α3 integrin (red) and L1CAM (green) are expressed within basal epithelial cells at and behind the leading edge; L1CAM but not α3 integrin also stains the intraepithelial corneal nerves (ICNs) (Fig. 2). βIII tubulin (magenta) staining reveals fewer axons near the leading edge in W corneas; the diffuse staining for βIII tubulin (*) observed in W corneas at the leading edge indicates axon degradation. Axons near the leading edge of the MW corneas appear thicker and more abundant. Representative cross-sectional views generated from 3D confocal images confirm that L1CAM and βIII tubulin co-localize on axons and that more axons are present near the leading edge in the MW compared to W corneas. As shown in Fig. 2, the leading edge in the wounded (W) cornea has 3 L1CAM+ βIII tubulin+ axons whereas the MW leading edge shows 6 axons (arrows). L1CAM and α3 integrin localization increases in basal cells located 3–4 cells behind the leading edge in both W and MW corneas (arrowhead).

### Debridement wounding with and without MMC treatment leads to significant changes in gene expression

The data presented above show that MMC treatment at the time of injury delays migration and enhances axon extension at the leading edge. Yet, MMC treatment of unwounded corneas disrupts axons 18 hr later. To gain insight into the mechanisms underlying the MMC induced delay in sheet migration and its impact on corneal sensory axons, we performed RNA-seq using RNA isolated from the corneal epithelium harvested by limbal to limbal debridement and from stromal buttons trimmed to remove limbal rims. Four variables are assessed: control corneas (C), corneas wounded by 1.5 mm debridement and sacrificed 18 hr after injury (W), corneas treated with MMC at the time of wounding and sacrificed 18 hr after injury (MW), and control corneas treated with MMC 18 hr prior to sacrifice (MC). The 18 hr time point allows us to identify genes up and down regulated during reepithelialization and reinnervation of the ICNs. For each variable, four independent samples are used (see methodology). Fold changes and q values are calculated for the following comparisons: W:C, MC:C, MW:MC, and MW:W for the epithelium and stroma. The heat map generated from these analyses is shown in Supplementary Figure 1 and the numbers of genes up and down regulated 2-fold or greater are shown in Supplementary Table 2.

Supplementary Figs 2–5 show the five top GO terms and IPA analyses for the 4 different comparisons performed. Using the ontology terms identified in these analyses, we generated lists showing the fold changes observed in representative 2-fold up or downregulated epithelial and stromal genes with statistical significance indicated by blue text (Supplementary Table 3).

**Figure 3:**
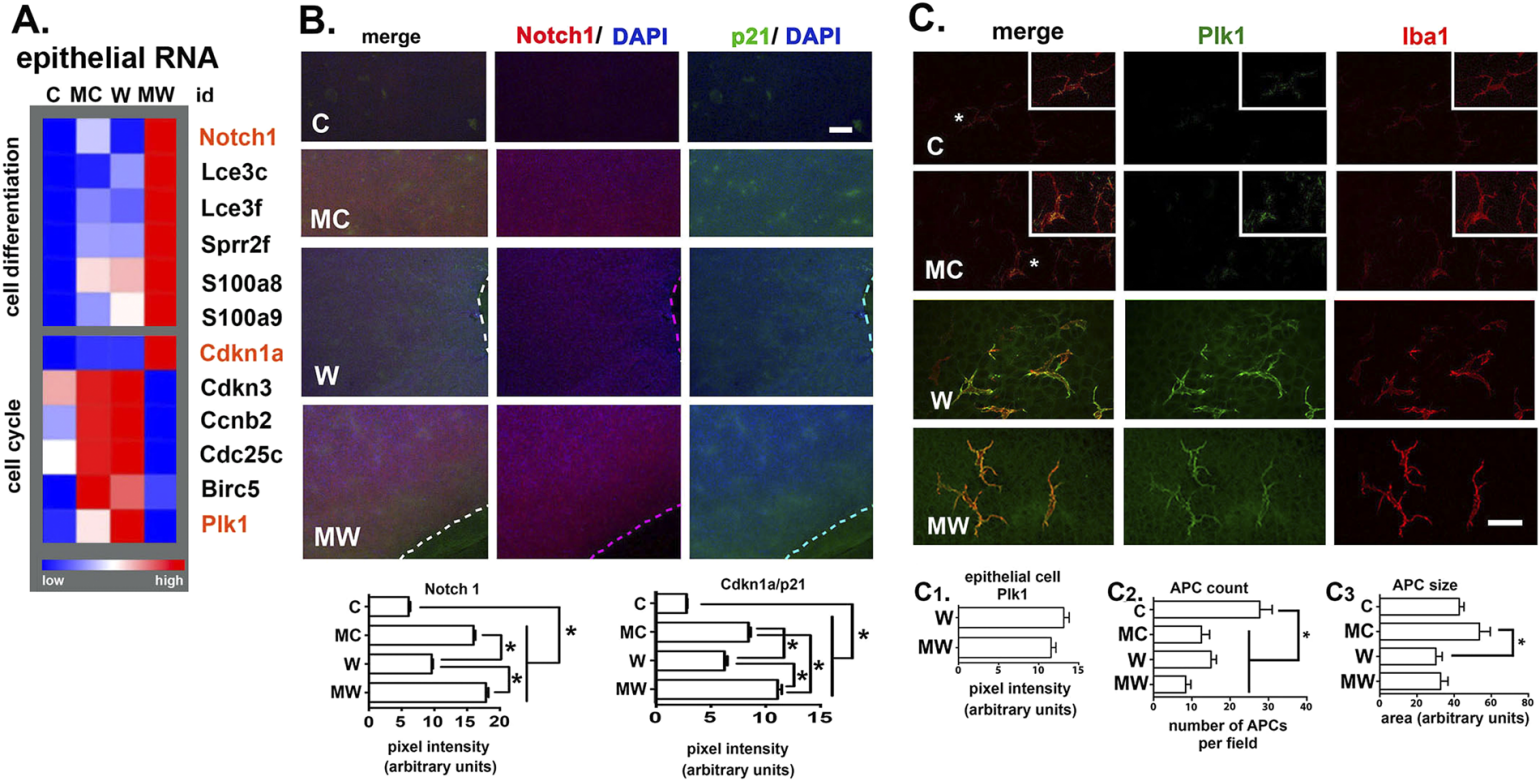
MMC treatment alters expression of cell differentiation and cell cycle genes in the cornea. (**A**) A heat map generated from RPKM values from RNA-seq data shows changes in gene expression of several representative cell differentiation and cell cycle genes. Details on the number of corneas used to obtain these data are presented in the methods section. (**B**) The localization of Notch1 (red) and Cdk1a/p21 (green) are shown in whole flat mount corneas for C, MC, W, and MW corneas imaged en face. Data are quantified below. Significance for Notch1 and Cdk1a/p21 was determined using the non-parametric Kruskal-Wallis test for groups with different standard deviations; groups with p values less than or equal to 0.05 are considered significant. Mag bar = 50 μm. (**C**) The localization and expression of Plk1 (green) is shown in flat mounted C, MC, W and MW corneas imaged en face showing the corneal periphery where Plk1 staining is maximal. Staining is also shown using an antibody against the APC marker Iba1 (red). Insets for C and MC show images for control corneas with colors enhanced to show cell shapes. Data are quantified for Plk1 expression in the epithelial cells surrounding the APCs (C1), for the total number of APC per field (C2), and for the APC size (C3). Significance for Plk1 intensity was determined using the unpaired t test, APC cell number (C2) and size (C3) was determined using the non-parametric Kruskal-Wallis test for groups with different standard deviations; groups with p values less than or equal to 0.05 are considered significant. Magnification bar in C = 30 μm.

**Figure 4:**
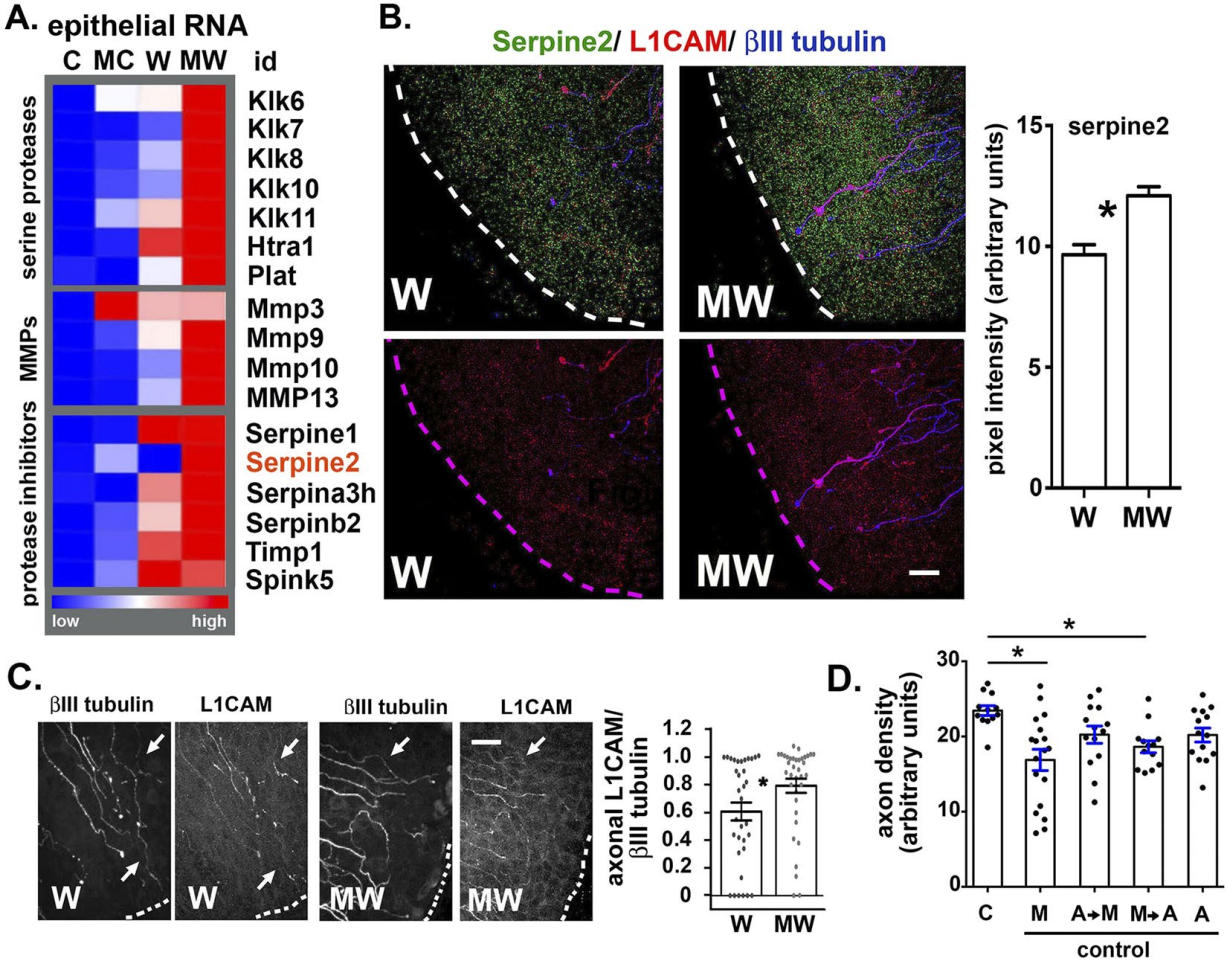
Wounding and MMC treatment alter expression of proteases and protease inhibitors in the cornea. (**A**) A heat map generated from RPKM values from RNA-seq data for C, MC, W, and MW shows changes in gene expression for several serine proteases and for protease inhibitors of the serpin family and for TIMP1. (**B**) Representative en face images showing the corneal sensory nerves of W and MW corneas stained to reveal the localization of serpine2 (green), L1CAM (red), and βIII tubulin (blue); bar graphs to the right show quantification of the serpine2 data. (**C**) Representative confocal images were captured for W and MW corneas stained for L1CAM and βIII tubulin and NeuronJ was used to trace axons for βIII tubulin and then for L1CAM. L1CAM/ βIII tubulin ratios were determined for 36 W and 34 MW individual axons near the leading edges of 4 different corneas for each variable. Bar graphs on the right show quantification. Significance was determined using the non-parametric Kruskal-Wallis test for groups with different standard deviations; groups with p values less than or equal to 0.05 are considered significant. (**D**) Unwounded corneas were treated with MMC alone (M), aprotinin followed by MMC (AM), MMC followed by aprotinin (MA), and aprotinin alone (A) and after 18 hr fixed, stained with antibodies against βIII tubulin, and Sholl analysis performed. Significance was determined by one-way ANOVA; p values less than or equal to 0.05 are considered significant. Magnification bars in B and C = 30 μm.

**Figure 5:**
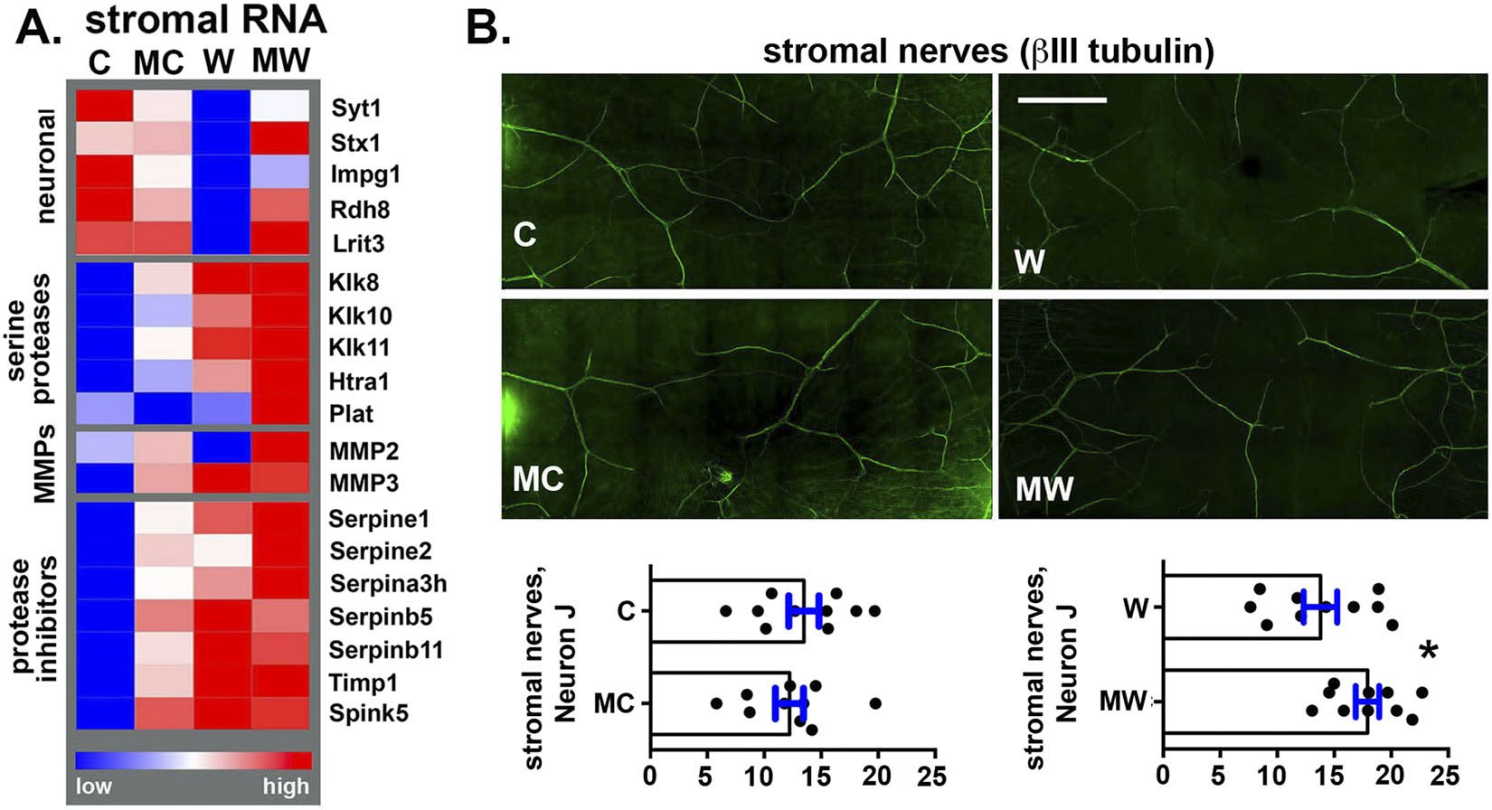
MMC treatment prevents wound-induced stromal nerve degeneration. (**A**) A heat map generated from RPKM values from RNA-seq data from stromal RNA isolated from C, MC, W, and MW corneas shows changes in gene expression for several neuronal-related, serine proteases, and protease inhibitor genes. (**B**) Representative confocal en face projection images from C, MC, W, and MW corneas stained to reveal the localization of βIII tubulin within corneal stromal nerves are presented. NeuronJ was used to quantify stromal neurons. 10 corneas from 5 mice were assessed for each variable; quantification is shown on the bottom panel. The presence of an intact corneal epithelium with βIII tubulin+ ICNs in C and MC corneas generates increased background staining and difficulty imaging stromal nerves compared to W and MW corneas which have no corneal epithelium at the corneal center and fewer ICNs in the periphery. As a result, comparisons are limited to C vs MC and W vs MW using unpaired t tests. There were fewer stromal nerves in W than MW. Magnification Bar in B = 100 μm.

### MMC treatment of wounded corneas increases Cdkn1a/p21 and apoptosis signaling and decreases expression of genes mediating cell cycle progression in the epithelium

In fibroblasts, MMC treatment can induce cell cycle arrest at G2/M^20,21^. RNA-seq of epithelial RNA indicates that MMC treatment of wounded corneas alters expression by 2-fold or greater compared to untreated wounded corneas (MW:W) of 238 genes with 155 genes upregulated and 83 genes downregulated (Supplementary Table 1). GO analyses for MW:W genes in the epithelium show upregulation of genes that regulate keratinocyte differentiation and IPA analyses reveal upregulation for genes involved in p53 signaling and apoptosis and downregulation of genes involved in regulating polo-like kinases and G2 checkpoint regulation (Supplementary Figure 5).

Heat maps in Fig. 3A show differences in expression of genes regulating cell differentiation and cell cycle progression for epithelial RNA from C, MC, W, and MW corneas. Heat maps are generated using mean RPKM values; Supplementary Table 3 shows mean fold change values for W:C, MC:C, and MW:W for genes in Fig. 3A to show those that are statistically significant. The increased expression of Notch1, Lce3c, Lce3f, Sprr2f, and Cdkn1a/p21 in MW compared to W epithelium are significant as is the decreased expression of Cdkn3, Ccnb2, Cdc25c, and Plk1. Wounding the corneas or treating control corneas with MMC 18 hr prior to sacrifice also increases expression of several cell differentiation genes shown in Fig. 3A. In contrast to wounded corneas, MMC treatment of control corneas increased expression of 5 of the 6 cell cycle genes. Thus, while a single topical treatment of MMC to the cornea impaired expression of mRNAs for proteins that promote cell cycle progression in migrating epithelial cells, it enhanced their expression in quiescent cells. These changes in cell cycle genes are accompanied by increased expression of genes that regulate epithelial cell differentiation after wounding and MMC treatment.

We next use confocal imaging to localize Cdkn1a/p21 and Notch1 proteins (Fig. 3B). As suggested by RNA-seq data, both p21 and Notch1 are significantly increased in W and MW corneas compared to C and MC corneas. While RNA-seq data shows no change in expression of Cdkn1a/p21 and Notch1 mRNAs after wounding (compare C to W in Fig. 3A), expression of both proteins is increased significantly. MMC increases expression of both proteins regardless of whether corneas are wounded.

Plk1 is a cell cycle regulated kinase that controls mitotic entry after G2^22^. Within C, MC, W, MW corneas, we found that Plk1 localization is enriched at the corneal periphery within wounded corneal epithelium and within Antigen Presenting Cells (APCs) that stain positive with antibodies against ionized calcium-binding adapter molecule1 (Iba1) (Fig. 3C). Iba1 is expressed on microglia and APCs and increases upon their activation^23,24^. Both Iba1 and Plk1 are increased in localization in APCs in W and MW corneas; Plk1 protein expression is not significantly reduced at the periphery in MW compared to W corneas (Fig. 3C1). Wounding (W, MW) or MMC treated unwounded corneas (MC) significantly reduces the number of APCs present at the periphery (Fig. 3C2). These data indicate that APCs respond to MMC treatment similarly whether the epithelium is intact or injured. While no significant differences in the sizes of APCs are seen between C and MC corneas and between W and MW corneas, APCs are larger in unwounded (C, MC) compared to wounded (W, MW) corneas (Fig. 3C3).

### Debridement wounding and MMC treatment increase serine protease, MMP, and protease inhibitor gene expression

The increased localization of Cdkn1a/p21 and Notch1 coupled with reduced localization of Plk1 in corneal epithelium of MW corneas shown by confocal imaging confirm RNA-seq data and indicate that MMC treatment of wounded corneas impacts corneal epithelial cell cycle progression as well as corneal epithelial cell differentiation. We next analyzed the epithelial RNA-seq data to identify RNAs for genes coding proteins that are secreted; differences in numerous genes encoding serine proteases and MMPs in C, MC, W, and MW corneas (Fig. 4A) are seen.

Expression of RNAs for several serine protease inhibitors (PIs) is also increased in MC, W, and MW corneas (Supplementary Table 3). When we compare W:C (Fig. 4A and Supplementary Table 3), four PIs are up regulated significantly in the wounded epithelium (Serpina3h, Serpinb2, Timp1, and Spink5). Serpine2 (glial derived nexin) is the only PI upregulated significantly (17 fold) in the MW:W corneal epithelium. Immunofluorescence was performed to localize serpine2, L1CAM, and βIII tubulin in W and MW corneas. We found serpine2 significantly increased in the cytoplasm of corneal epithelial cells at the leading edge in the MW compared to W corneas (Fig. 4B).

L1CAM:L1CAM and L1CAM:integrin cell:axon adhesions stabilize axons within the epithelium^25,26^. The epitope recognized by the L1CAM antibody is within its extracellular domain; discontinuous staining for L1CAM along axons indicates shedding from axonal and/or epithelial cell surfaces. Shedding is mediated by serine proteases and MMPs^27–29^ and differences in L1CAM loss from axons indicates differences in extracellular proteolysis. To quantify the extent of L1CAM shedding, we analyzed images from 4 different W and MW corneas showing βIII tubulin and L1CAM at the leading edge and traced axons using NeuronJ to determine the ratio of L1CAM to βIII tubulin (Fig. 4C). The majority of axons show both proteins localize along the length of axons and yield L1CAM to βIII tubulin ratios of 1. This ratio is significantly higher for MW than for W. These data indicate that MMC treatment of wounded corneas enhances retention of L1CAM on axons near the leading edge.

MMC treatment may promote reinnervation after debridement by maintaining L1CAM:L1CAM and/or L1CAM:α3 integrin cell:axon adhesion. Blocking proteinase activity after wounding prevents reepithelialization and sheet movement^30^. MMC treatment of unwounded corneas also alters protease and protease inhibitor RNA expression; axon density is significantly reduced 18 hr after MMC treatment (see Fig. 1D). To confirm that proteases impact axon morphology after MMC treatment, unwounded corneas were treated with the serine protease inhibitor- aprotinin for 2 minutes prior to or 2 minutes after MMC treatment; unwounded corneas were also treated with aprotinin alone or with MMC alone. Mice were sacrificed 18 hr later and corneal axon densities determined (Fig. 4D). Treating unwounded corneas with aprotinin before MMC reduces MMC-induced loss of axon density; aprotinin treatment after MMC did not preserve axon density but did have a partial effect. These experiments confirm that serine protease activation induced by MMC in unwounded corneas can be partially blocked with a serine protease inhibitor.

### MMC attenuates stromal nerve loss after debridement wounding

In the stroma, GO and IPA analyses for MW:W (Supplementary Figure 5) yield terms showing increased expression of genes involved in neurogenesis or nerve function. Of the 764 genes up regulated 2-fold or greater in the MW:W stroma, 443 (43%) are down regulated in the W:C stroma (Supplementary Figure 5). As a result, GO and IPA terms regulating nerve development and signaling are increased in the MMC-treated wounded stroma (MW:W).

Figure 5A is a heat map showing differences in stromal RNA expression for neuronal-related genes as well as for proteases and protease inhibitors. Shown are 5 neuronal-related RNAs (Syt1, Stx1b, Impg2, Rdh8, and Lrit3) reduced in expression by more than 2-fold in wounded corneas; 3 of those 5 (Stx1b, Rdh8, and Lrit3) are increased 2-fold or more in the MW:W comparison. These genes have not been evaluated outside the retina and CNS. In Fig. 5A, we show that W, MC, and MW corneal stroma increase expression of serine proteases and protease inhibitors. The changes seen in 4 kallikreins and MMP3 as well as the serine protease inhibitors serpine1 and Spink5 and MMP inhibitor TIMP1 are statistically significant in W:C and MC:C but not MW:W corneas (Supplementary Table 3). To determine whether these differences in protease and protease inhibitor gene expression impact stromal nerves, we next imaged stromal nerves of wounded corneas and found that their arborization, assessed using NeuronJ, is not significantly altered in MC compared to C corneas but is greater in the MW compared to W corneas (Fig. 5B). Since ICNs branch from stromal nerves, a reduction in stromal nerves will delay ICN reinnervation.

### MMC treatment at the time of trephine injury delays reinnervation

**Figure 6:**
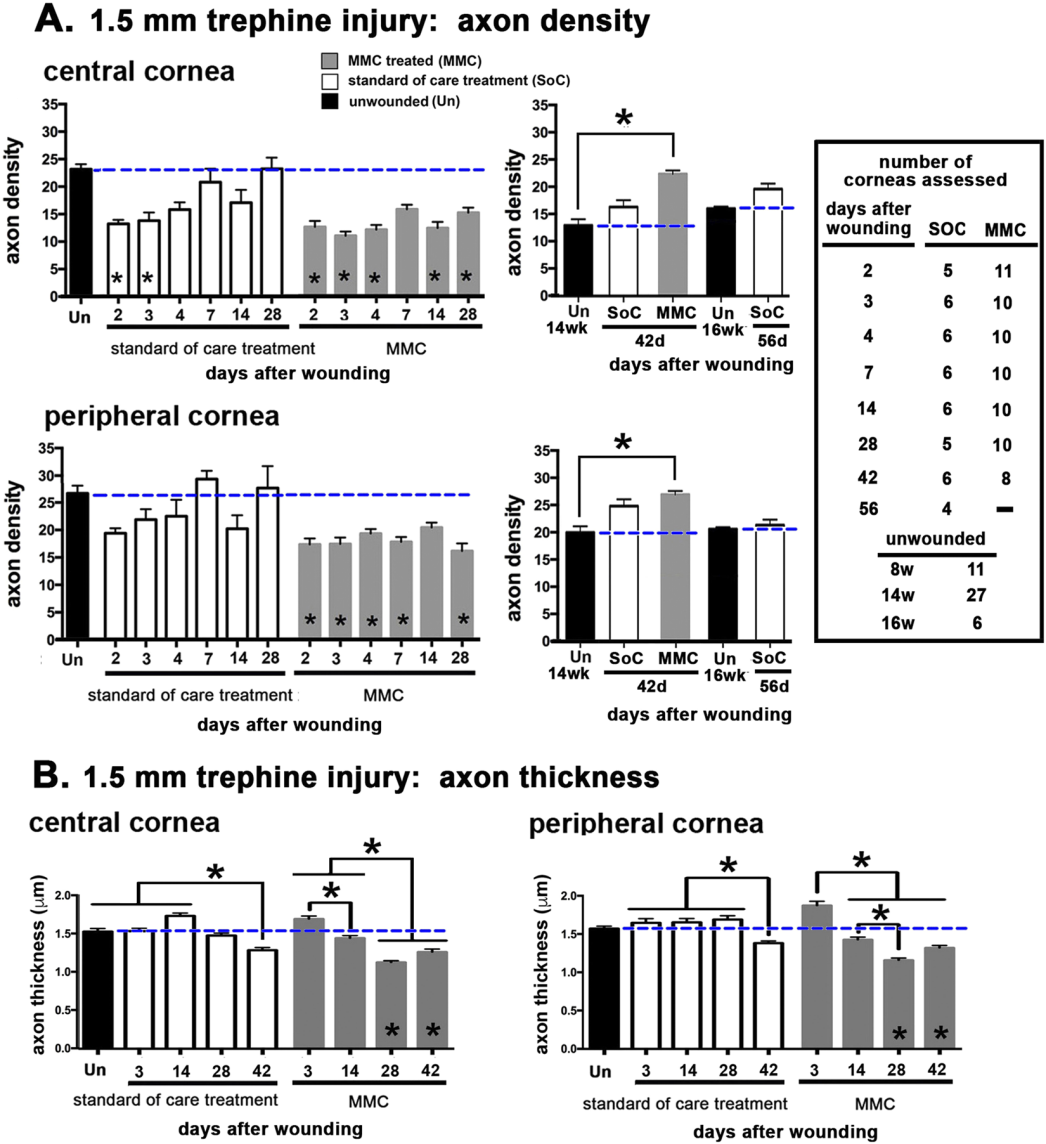
MMC treatment transiently delays INT reinnervation after trephine-only injury. (**A**) Mice were subjected to 1.5 mm trephine injury and sacrificed at the times indicated; a single treatment of MMC was applied to MMC treated corneas; control corneas were subjected to routine standard of care (SOC) conditions. Mice were sacrificed at the times indicated. Corneas were stained with antibodies against βIII tubulin, flat mounted, imaged, and Sholl analysis performed. The number of corneas assessed for each time point and variable are indicated. For times longer than 28 days, age matched unwounded corneas were also assessed. (**B**) Axon thickness was also assessed after trephine injury. For axon density and thickness, significance was determined using the non-parametric Kruskal-Wallis test for groups with different standard deviations; groups with p values less than or equal to 0.05 are considered significant. The table shown in the inset indicates the number of corneas used for each time point and variable assessed.

We show above that MMC treatment of unwounded corneas disrupts the ICNs by increasing protease gene expression and activity within the epithelium but has no impact on stromal nerves. Yet, MMC treatment at the time of debridement injury stabilizes both ICNs and stromal nerves and preserves L1CAM localization on reinnervating ICNs by a mechanism that involves changes in proteinase activity and protein degradation extracellularly. These data predict that treating corneas with severed or crushed nerves with MMC without inducing cell migration would delay reinnervation by activating proteases and destabilizing axons. In the trephine injury model, a dulled 1.5 mm trephine is placed on the corneal surface and rotated to generate an impression on the epithelial surface; this injury kills the corneal epithelial cells under the trephine blade and severs or crushes 50% of the INTs within the 1.5 mm area defined by the trephine^31^. We next compare axon density during reinnervation after our SOC and MMC treatment at 2, 3, 4, 7, 14, and 28 days after trephine injury (Fig. 6A). In SOC-treated trephine injured corneas, axon density is significantly reduced at the corneal center but not the periphery at 2 days and recovers to control levels at the corneal center by 4 days. In the MMC treated corneas, axon density is significantly reduced at the periphery at all time points except 14 days and at the center at all time points except 7 days. We next compare axon density in age-matched corneas at 42 days after trephine injury with MMC treatment; we find that axon density recovers at both the corneal center and periphery (Fig. 6A). The delayed recovery of axon density after MMC treatment of trephine-injured corneas is accompanied by significantly reduced thickness of axons (Fig. 6B). These experiments confirm that while MMC treatment enhances reinnervation after debridement injury where reepithelialization takes place, MMC treatment of injuries that do not require reepithelialization transiently delays sensory axon recovery.

## Discussion

### The impact of MMC on corneal epithelial homeostasis *in vivo* depends on whether it is applied to quiescent or migrating corneal epithelial cells

Here we show that a single topical MMC treatment alters gene expression in both quiescent and migrating epithelial cells. By slowing the rate of epithelial cell migration and reducing cell cycle gene expression after debridement injury, MMC treatment enhances sensory axon recovery and attenuates stromal nerve loss after wounding via a mechanism that involves increased expression of the protease inhibitor serpine2 by injured corneal epithelial cells which retains L1CAM on ICN surfaces.

Topical MMC treatment of uninjured corneas also increases protease gene expression and activity, and transiently disrupts sensory nerves. If similar changes in epithelial cell gene expression and sensory axon stability occur after MMC treatment of trephine wounded corneas where the corneal epithelium remains on the ocular surface, axon recovery would be delayed, which is what we observe. In addition to altering extracellular proteostasis, application of MMC to unwounded corneas also results in a significant increase in expression of epithelial mRNAs associated with cell cycle progression (Cdkn1a/p21, Ccnb2, Cdc25c, and Plk1) whereas application of MMC to wounded corneas significantly decreases expression of these mRNAs with the exception of Cdkn1a/p21.

MMC treatment of cultured primary and tumor cells leads to cell cycle arrest at the G2/M checkpoint^20,21^ and we did not expect that mRNAs for genes for cell cycle proteins would be up regulated after MMC treatment in control corneas. Proliferation of basal epithelial cells forces neighboring cells to differentiate and become displaced apically leading to their terminal differentiation; the increased expression of genes for cell differentiation markers indicates this is occurring in the MC corneal epithelium. The changes seen in axon density in MC and superficially wounded (trephine injured) corneas are transient as indicated by the eventual recovery of axon density 42 days after trephine injury. Having obtained a molecular level understanding of the impact of MMC on gene expression, we better understand how it reduces non-regenerative (fibrotic) wound repair.

### MMC treatment at the time of injury preserves stromal nerves

The signals elicited by severing of the ICNs by epithelial debridement are transmitted to the stromal axons both directly and indirectly by the swelling of the stroma after injury. Here we show a significant loss of stromal nerves after debridement which is accompanied by a decrease in the expression of numerous neuronal-associated genes within the stroma including synaptotagamin1 (Syt1), interphotoreceptor matrix proteoglycan 2 (Impg/Sparcan), retinol dehydrogenase-8 (Rdh8), Leucine-Rich Repeat, and immunoglobulin-like and transmembrane domains 3 (Lrit3) and that MMC prevents this downregulation. Whether these genes are expressed by non-myelinating Schwann cells or stromal cells and translated into proteins has not been determined. Further mining of these RNA-seq data and additional RNA-seq studies on corneas with deeper stromal wounds will lead to new insights into the mechanisms behind MMC’s ability to improve regenerative repair and suppress fibrosis within resident stromal cells.

### Corneal epithelial cells function as non-myelinating Schwann cells for the corneal sensory nerves

The epithelium lacks neural crest derived Schwann cells like those surrounding the thicker stromal nerves that branch and give rise to the ICNs. To maintain and support the ICNs, the corneal epithelial cells perform many Schwann cell functions. Since ICNs are localized primarily between basal cells, displacement of differentiating cells apically mechanically destabilizes them. ICNs continuously grow, sever, and regrow throughout life^2^. Aging reduces ICN density in humans^32–35^ and mice^3,31,36^. Aging leads to reduced expression of genes by corneal epithelial cells that promote axonal elongation in the mouse^3^. As Schwann cell surrogates, corneal epithelial cells express numerous Schwann cell proteins. In addition to L1CAM and serpine2, originally referred to as glial derived nexin, we show that the epithelium expresses nerve growth factor, synaptotagamins 12 and 16, GDNF, and the intermediate filament protein nestin (Supplementary Table 2).

The mouse L1CAM molecule has two RGD domains that allow it to bind to integrins^25,37^. The extracellular domain of L1CAM is shed in response to axon injury and/or inflammatory cues^26–29,38^. Shed L1CAM and Sema3a together modulate the ability of the Sema3a receptor complex to mediate axon targeting^38^. Mutations in L1CAM lead to neurologic anomalies^39,40^. L1CAM is expressed on axons and Schwann cells where it mediates axon outgrowth^25,41^. Along with neuropilins and plexins, L1CAM is a component of the Sema3a receptor complex^42^; Sema3a plays a role in sensory axon reinnervation in the mouse cornea^43,44^.

We show here that L1CAM mRNA and protein are expressed by corneal epithelial cells and the protein co-localizes with α3 integrin within corneal epithelial basal cells and with βIII tubulin within corneal sensory axons. By inducing alterations in protease and protease inhibitor gene expression in the epithelium after wounding, MMC reduces L1CAM shedding on reinnervating corneal sensory axons after debridement injury to the cornea. Previously, we showed that MMC applied topically soon after reepithelialization is complete, promotes stable reinnervation over time after debridement injury^6^. Here we show that MMC enhances axon reinnervation despite delaying reepithelialization.

While RNA-seq data show that wounding increases epithelial expression of several serpin family members, serpine2 is the only serpin upregulated significantly in both MMC treated control and wounded epithelia. Studies of peripheral nerve regeneration using the sciatic nerve crush model have shown that serpine2 mRNA is upregulated during axonal regeneration^45^. Serpins are family of serine protease inhibitors^46,47^ that include plasminogen activator inhibitor 1 (PAI-1 or serpine1) and serpine2 [glial derived nexin or protease nexin 1 (PN-1)]. Both serpins inhibit tPA, uPA, and other serine proteases capable of cleaving L1CAM. After secretion, serpine1 enters the serum, while serpine2 binds to glycosaminoglycans within the basement membrane and stroma staying localized within tissues^48^. As such, serpine2 can bind to and inhibit the activity of serine proteases secreted by migrating epithelial cells including kallikreins. The fact that serpine2 mRNA and protein are upregulated in MW corneal epithelium, L1CAM is retained on the surfaces of regenerating axons in MW corneas, and a serine protease inhibitor retains axons on MC corneas, together provide strong evidence for MMC facilitating reinnervation by altering the cleavage and turnover of extracellular proteins including the extracellular domains of cell membrane proteins such as L1CAM.

### MMC: a tool to use to understand corneal wound repair

MMC is used in ophthalmology to reduce scarring after glaucoma^49^ and refractive surgery^7,9,10,50^. It also reduces scarring after vocal cord^51,52^ and nasal surgery^53^. MMC is used to treat pterygium and ocular tumors^54,55^. While concerns over long term effects of MMC use have been noted in the literature^56^, it remains in clinical use.

MMC treatment of the cornea alters expression of numerous mRNAs and proteins in the epithelium including proteases (kallikreins and MMPs) and protease inhibitors including serpine2. In the wounded corneal epithelium, these changes slow reepithelialization but positively impact corneal epithelial cell:axon interaction and lead to reduced shedding of L1CAM and enhanced reinnervation. In the stroma, after debridement, MMC treatment attenuates the loss of stromal nerves due to injury. While MMC treatment of quiescent corneal epithelium induces protease and protease inhibitor RNA expression and reduces ICN density, it has no impact on stromal nerve integrity. It does, however, increase epithelial cell expression of RNAs that positively regulate cell cycle progression and differentiation. These data extend our understanding of sensory axon:corneal epithelial cell interaction during homeostasis and in response to injury as well as support a role for the epithelial cells in the ability of MMC to suppress scarring and regulate fibrosis.

## Electronic supplementary material

Supplementary Data excluding Supplemenary Table 2

Supplementary Table 2
