# Supplementary material for: Molecular basis of Mitomycin C enhanced corneal sensory nerve repair after debridement wounding
Source: Sci Rep. 2018 Nov 16;8:16960. doi: 10.1038/s41598-018-35090-3 (PMC6240058; doi:10.1038/s41598-018-35090-3)

Supplementary Data for:
Molecular basis of Mitomycin C enhanced corneal sensory nerve repair after debridement wounding

Mary Ann Stepp[1,2*], Sonali Pal-Ghosh[1], Gauri Tadvalkar[1], Luowei Li[3], Stephen R. Brooks[4], and Maria I. Morasso[5]

[1] Department of Anatomy and Cell Biology, George Washington University School of Medicine and Health Sciences, Washington DC 20037, USA

[2] Department of Ophthalmology, George Washington University School of Medicine and Health Sciences, Washington DC 20037, USA

[3] Laboratory of Cancer Biology and Genetics, NCI, NIH, Bethesda, MD 20892, USA

[4] Biodata Mining and Discovery Section, National Institute of Arthritis and Musculoskeletal and Skin Diseases, National Institutes of Health, Bethesda, MD, 20892, USA

[5] Laboratory of Skin Biology, National Institute of Arthritis and Musculoskeletal and Skin Diseases, National Institutes of Health, Bethesda, MD, 20892, USA

***communicating author:**
Mary Ann Stepp
Department of Anatomy and Cell Biology
George Washington University School of Medicine and Health Sciences, Washington DC 20037
mastepp@gwu.edu
1-202-994-0557

Supplementary Table and Figure Legends:

Supplementary Table 1: Total number of genes whose expression is altered up or down 2-fold.

Supplementary Table 2: Gene Lists for 2-fold up and down regulated genes. See attached Excel file

Supplementary Table 3: Representative up and down regulated genes from the corneal epithelium and stroma. The gene lists generated and presented in Supplemental Table 2 and the results of the GO and IPA analyses presented in Supplemental Figures 2-5 were used to generate representative lists of genes for pathways altered by wounding and MMC treatment within the corneal epithelium and stroma highlighting the fold changes seen in the following comparisons: W:C, MC:C, and MW:W. Blue text indicates that the fold changes observed are statistically significant.

Supplementary Figure 1: Heat map for epithelial and stromal RNA-seq data.

Supplementary Figure 2: Analysis of RNA-seq data from wounded compared to control (W:C) corneal epithelium and stroma. A. Gene Ontology terms. **B.** IPA terms.

Supplementary Figure 3: Analysis of RNA-seq data from unwounded corneas treated 18 hr prior to sacrifice with MMC compared to control (MC:C). A. Gene Ontology terms. **B.** IPA terms. **C.** Venn Diagram highlighting that most of the genes upregulated in the MC:C epithelium and stroma are upregulated in the W:C epithelium and stroma.

Supplementary Figure 4: Analysis of RNA-seq data from MMC treated 18 hr wounded compared to unwounded corneas treated 18 hr prior to sacrifice with MMC (MW:MC). A. Gene Ontology terms. **B**. IPA terms.

Supplementary Figure 5: Analysis of RNA-seq data from MMC treated 18 hr wounded corneas compared to untreated wounded (MW:W) corneal epithelium and stroma. A.

Gene Ontology terms. **B.** IPA terms. **C.** Venn Diagram showing that most of the genes downregulated in the W:C stroma are upregulated in the MW:W stroma.

Supplementary Table 1: Total number of genes whose expression is altered up or down 2-fold or more.

		W:C	MC:C	MW:W
epithelium	2x up:	621	234	156
	2x down:	484	23	84
	up+down:	1105	257	238
stroma	2x up:	1016	655	815
	2x down:	769	22	101
	up+down:	1785	677	916
enriched* stroma	2x up:	724	480	764
	2x down:	718	19	80
	up+down:	1442	499	844

* see RNA-seq in Methods section for description of of how stromal enriched gene lists were generated.

Supplementary Table 2: Gene Lists for 2-fold up and down regulated genes. Excel file

Supplementary Table 3 : Representative up and down regulated genes from the corneal epithelium and stroma

Epithelial RNA		W:C	MC:C	MW:W
Differentiation	Notch1	1	1.4	2.1
	Lce3c	13	3.7	3.2
	Lce3f	1.7	2	2.7
	Sprr2f	8.4	7.8	2.8
	S100a8	7	5.5	1.4
	S100a9	5.4	3.4	1.8
Cell Cycle Regulation	p21/Cdkn1a	1.2	1.3	2.7
	Cdkn3	1.3	1.2	-3.5
	Ccnb2	1.9	1.8	-3.2
	Cdc25c	1.6	1.5	-4.4
	Plk1	2.9	2	-3.7
Serine Proteases	Klk6	6.7	6.2	1.8
	Klk7	6.5	1.5	4.6
	Klk8	5.8	2.3	2.3
	Klk10	3.8	2.4	2.7
	Klk11	2.7	2	1.4
	Htra1	7.5	1.4	1.2
	tPA/PLAT	1.5	-1.1	1.4
MMPs	MMP3	2.1	5	1.6
	MMP9	6	2.2	1.6
	MMP10	3.5	1.3	2.7
	MMP13	27.2	2.3	2.3
MT-MMPs/ ADAMs	MMP14	1.2	-1.2	1
	MMP15	1.5	1.1	-1.5
	ADAM9	1.2	1	-1.1
	ADAM10	1.1	1.1	1.1
	ADAM17	-1.1	1	1.1
Protease Inhibitors	Serpine1	28	1.6	-1.2
	Serpine2	-1.2	4.9	17.1
	Serpina3h	4.6	-1.3	1.3
	Serpinb2	7	2.2	1.4
	Serpinb5	1.1	1	1.3
	Timp1	15.4	4	1.1
	Spink5	6.9	2.6	-1.1

Stromal RNA		W:C	MC:C	MW:W
Differentiation	Notch1	-1.3	1.5	3.5
	Lce3c	13.3	5.4	1.2
	Sprr2f	8.9	5.7	1.5
	S100a8	45.6	20.9	-1.9
	S100a9	55.7	22.4	-1.9
Cell Cycle Regulation	p21/Cdkn1a	1.4	1.5	2.8
	Cdkn3	6.7	4	-4.9
	Ccnb2	4.8	3.4	-2.5
	Cdc25c	4.4	2.9	-2.7
	Plk1	4.4	3.1	-1.9
Serine Proteases	Klk6	9.2	3.8	1.4
	Klk8	7.7	3.2	1
	Klk10	4	2	1.5
	Klk11	4.4	2.3	1.1
	Htra1	1.7.	1.3	1.3
	tPA/PLAT	1	-1.2	1.5
MMPs	MMP3	89.7	20.5	-1.6
	MMP9	1.3	-1.1	1.6
MT-MMPs/ ADAMs	MMP14	-1.1	-1.2	1.1
	MMP15	-1.6	-1.2	1.1
	ADAM9	-1.5	1	1.5
	ADAM10	1.1	1.2	1.1
	ADAM17	-1.2	1	1.4
Protease Inhibitors	Serpine1	2.9	2	1.3
	Serpine2	2.2	2.5	2.1
	Serpina3h	2	1.7	1.3
	Serpinb5	3	2.3	-1.3
	Timp1	80.6	14.1	-1.1
	Spink5	5.1	3.8	-1.2
Neuronal--related	Syt1	-3.8	-1.5	2.4
	Stx1b	-3.2	-1.3	4.4
	Impg2	-4.1	-1.5	2.3
	Rdh8	-4.7	-1.5	4.1
	Lrit3	-6.9	-1.8	5

Supplementary Figure 1

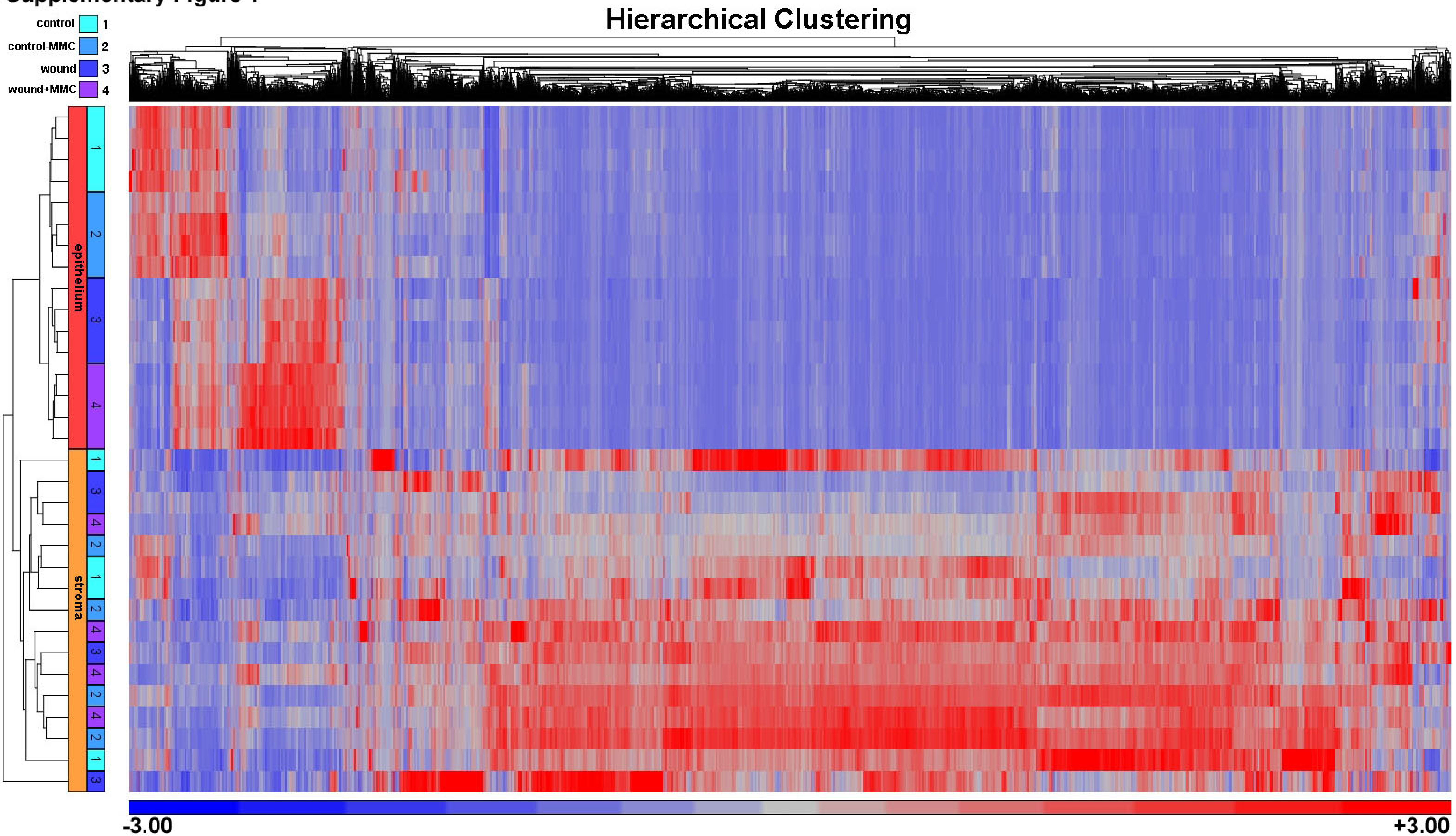

Supplementary Figure 2

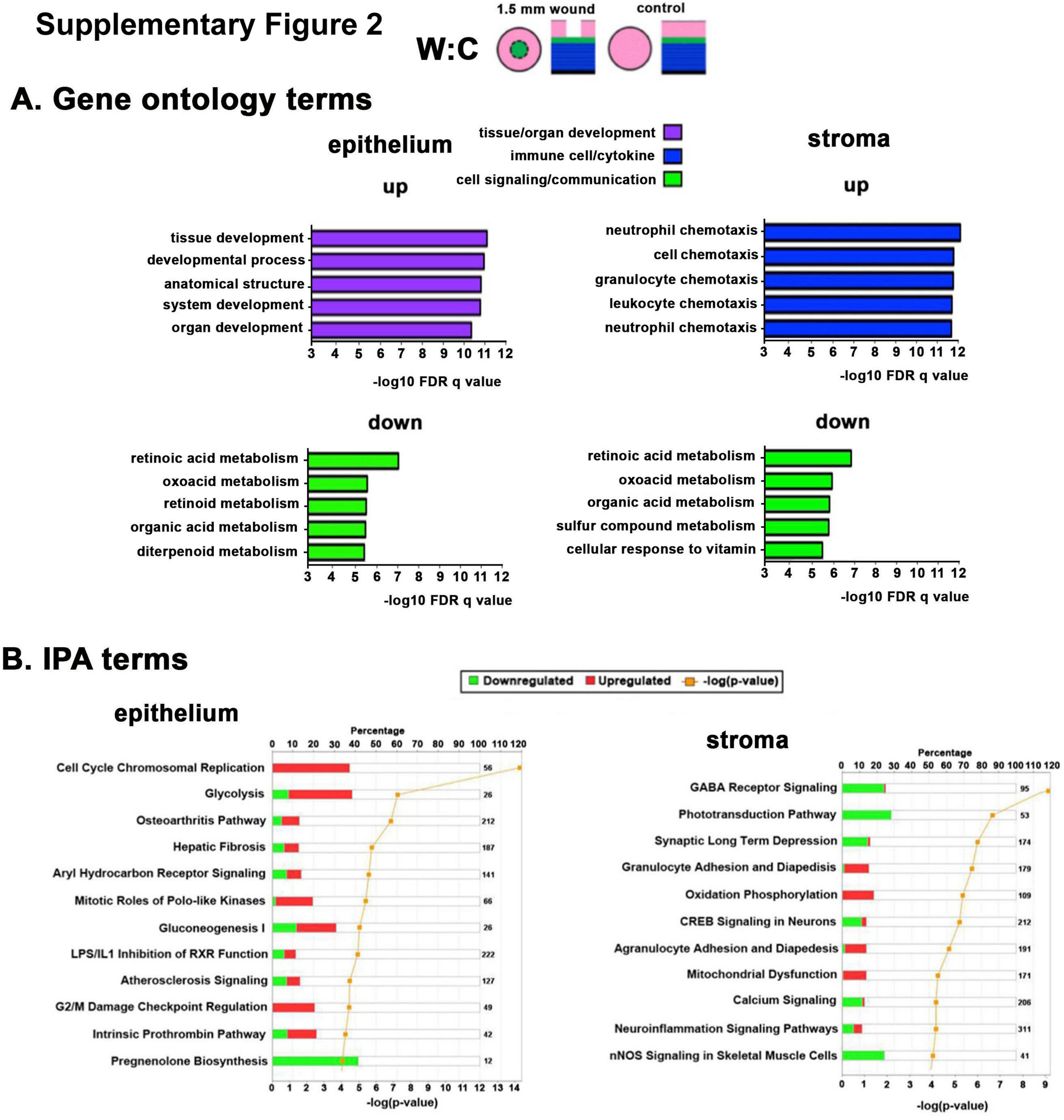

Supplementary Figure 3

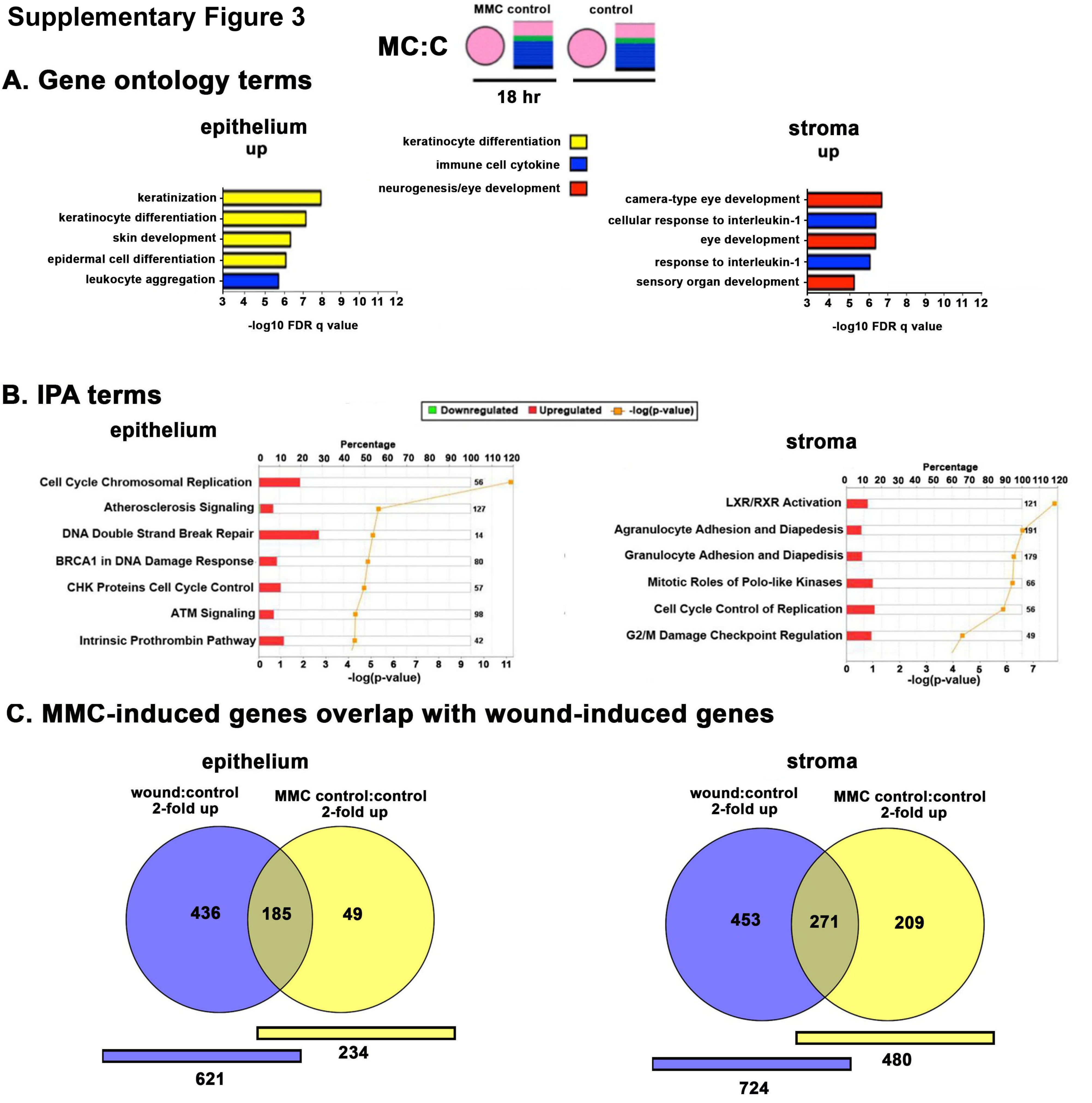

Supplementary Figure 4

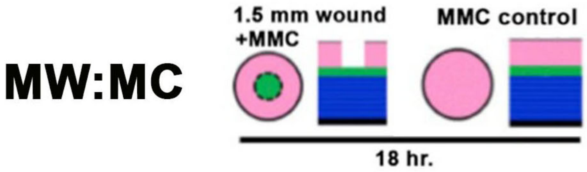

A. Gene ontology terms

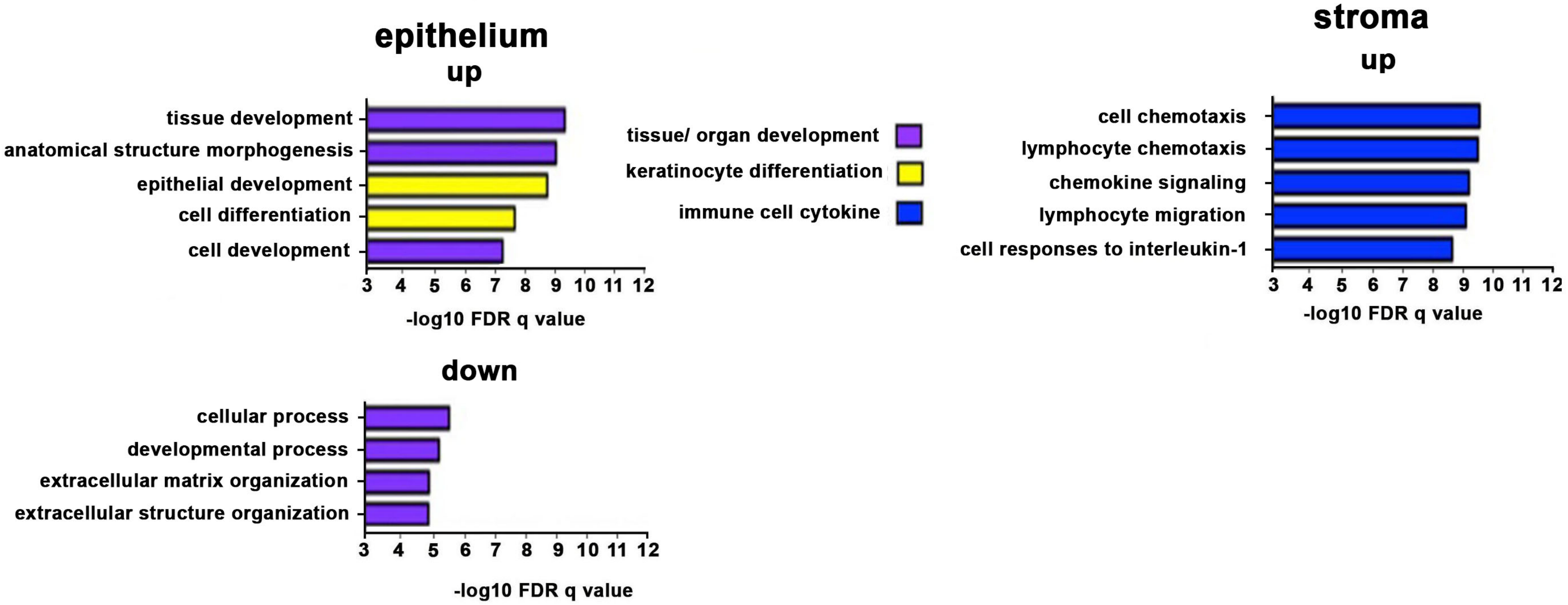

B. IPA terms

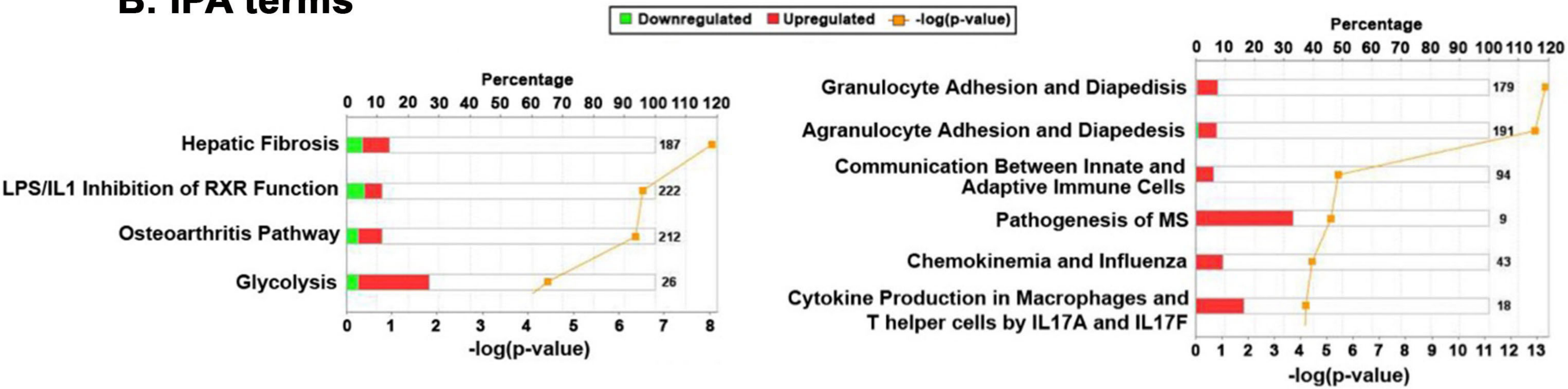

Supplementary Figure 5

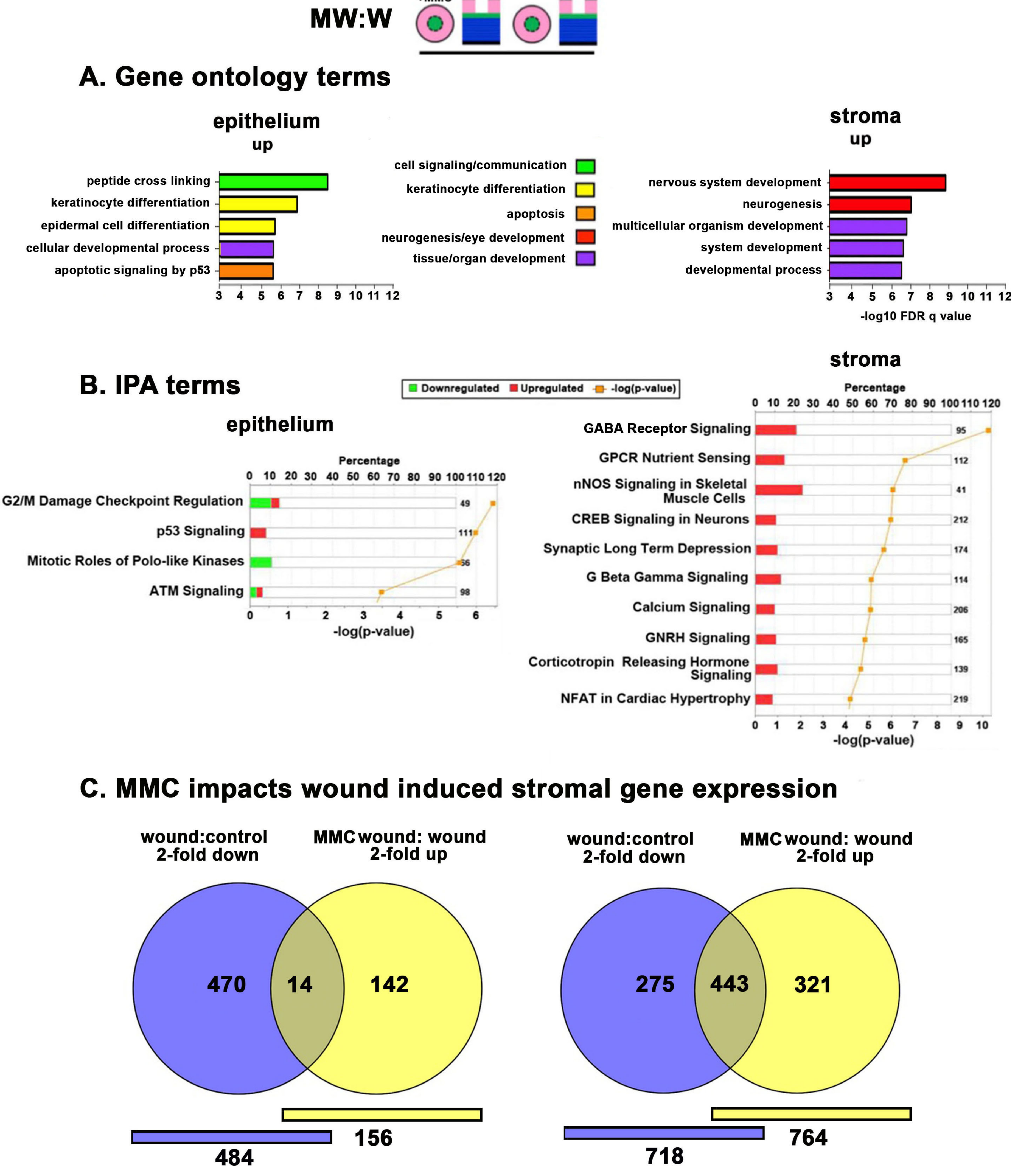

Supplement: Supplementary file 1 — Supplementary Data excluding Supplemenary Table 2 [file 41598_2018_35090_MOESM1_ESM.pdf]
